# Population Structure and Genetic Diversity Analyses Reveal Isolation That May Imperil the Northernmost Colony of the Endangered Australian Sea Lion

**DOI:** 10.1002/ece3.73038

**Published:** 2026-02-04

**Authors:** Vanessa Morris, Anthony Chariton, Robert Harcourt, Catherine E. Grueber, Isabelle Charrier, Holly Raudino, Kelly Waples, Roger Kirkwood, Simon D. Goldsworthy, Benjamin J. Pitcher

**Affiliations:** ^1^ School of Natural Sciences Macquarie University Sydney New South Wales Australia; ^2^ School of Life & Environmental Sciences The University of Sydney Sydney New South Wales Australia; ^3^ CNRS, Institut Des Neurosciences Paris‐Saclay Université Paris‐Saclay Saclay France; ^4^ Department of Biodiversity, Conservation and Attractions Kensington Western Australia Australia; ^5^ South Australian Research and Development Institute West Beach South Australia Australia; ^6^ School of Biological Sciences The University of Adelaide Adelaide South Australia Australia; ^7^ Taronga Conservation Society Australia Mosman New South Wales Australia

**Keywords:** climate change, connectivity, conservation, SNPs, vulnerability

## Abstract

Marine environments are experiencing rapid warming, substantially altering ecosystems. Populations at the edge of a species' range are more vulnerable to environmental change as they are first affected and may have limited dispersal opportunities. This vulnerability may be exacerbated in species with specialised foraging and breeding strategies. The Australian sea lion ( *Neophoca cinerea* ) is an endangered otariid species that breeds across a ~3000 km range in southern Australia. At the most north‐westerly edge of the species' distribution, Australian sea lions breed across multiple islands within the Houtman Abrolhos Archipelago, Western Australia, a tropical‐temperate location affected by marine heatwaves. This study aimed to examine the genetic structure and diversity of the Australian sea lions from the Houtman Abrolhos Archipelago compared to other populations in the species' range. One hundred and twenty‐five individuals, 19 from Houtman Abrolhos, were genotyped from 19 sample sites across Western Australia and South Australia. Our findings showed that individuals from the Houtman Abrolhos grouped into a single population, which was highly differentiated and had extremely low genetic diversity. The isolation and limited genetic variation of the Houtman Abrolhos Australian sea lion population suggest that it is extremely vulnerable to extirpation. Our study highlights the vulnerability of isolated populations of a species to rapid environmental change and stochastic events.

## Graphical abstract

Rapid warming of marine environments is threatening ecosystems, especially species at the edge of their range. This study analysed the genetic structure and diversity of Australian sea lion populations and found the northernmost population to be highly isolated with extremely low genetic variation. Our study highlights the vulnerability of isolated populations of a species to rapid environmental change and stochastic events.

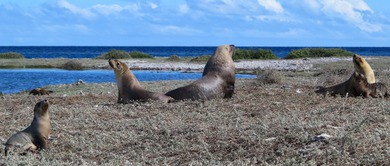

## 1 Introduction

In a rapidly warming world, organisms are shifting their geographic range in attempts to survive (Pecl et al. 2017; Pinsky et al. 2020). Marine environments are experiencing acute changes faster than terrestrial ecosystems (Poloczanska et al. 2016). Highly dynamic oceans are absorbing more than 90% of anthropogenically derived heat (Pachauri and Mayer 2015) and this is driving significant changes in coastal and oceanic marine systems. Marine heatwaves, periods of exceptionally high ocean temperatures (Laufkötter et al. 2020), have become more frequent, last longer and show increased intensities around the world (Elzahaby et al. 2021). Boundary currents that previously stabilised marine ecosystems are warming faster than the ocean average, inducing unpredictable weather systems that are altering ocean basin coastlines (Li et al. 2022). Marine organisms appear particularly vulnerable to these unstable and rapid changes, with many populations redistributing, fragmenting, contracting or going extinct (Smale et al. 2019). Even more acute are those which are central place foragers such as breeding seabirds and female otariids (fur seals and sea lions) (Nowicki et al. 2019; Boyd et al. 2017).

Understanding population structure is one way to identify potential vulnerabilities to environmental changes. Genetic markers can be used to determine population structure, genetic diversity and verify range shifts (Gervais et al. 2021). Populations that are highly differentiated and fragmented, often including those at the trailing edge of a species' range, are more vulnerable to changes as they have limited individuals to mix with and could contract within the species range or have nowhere else to extend to (Ramos et al. 2018). With limited connectivity, edge populations usually have low genetic diversity and high levels of inbreeding, relative to other populations across the species' range, increasing the former's risk of extinction (Frankham et al. 2002). Determining species' connectivity can assist with conservation actions by helping assess how resilient a range‐limited group of animals is to present and future climate change.

**FIGURE 1:**
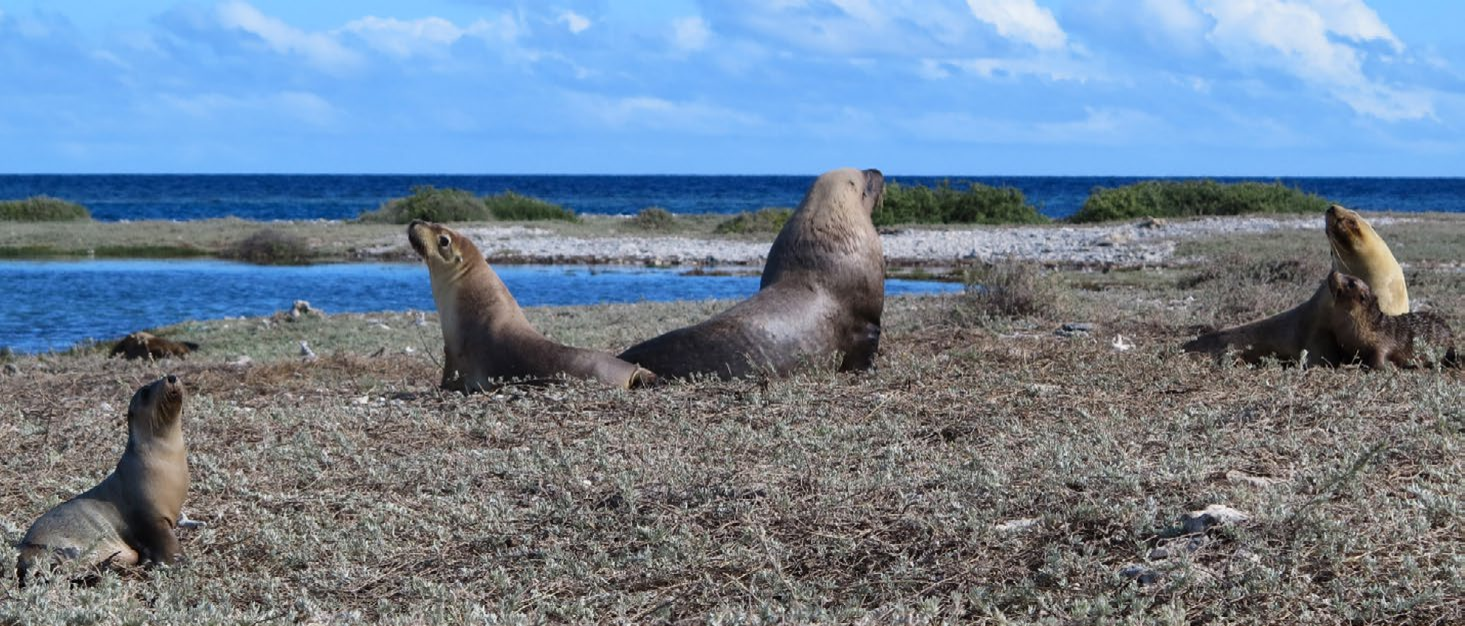
Australian sea lions at Gilbert Island in the Houtman Abrolhos Archipelago. From left to right, a juvenile, adult female, adult male and an adult female with a brown pup. Credit: Kelly Waples.

**FIGURE 2:**
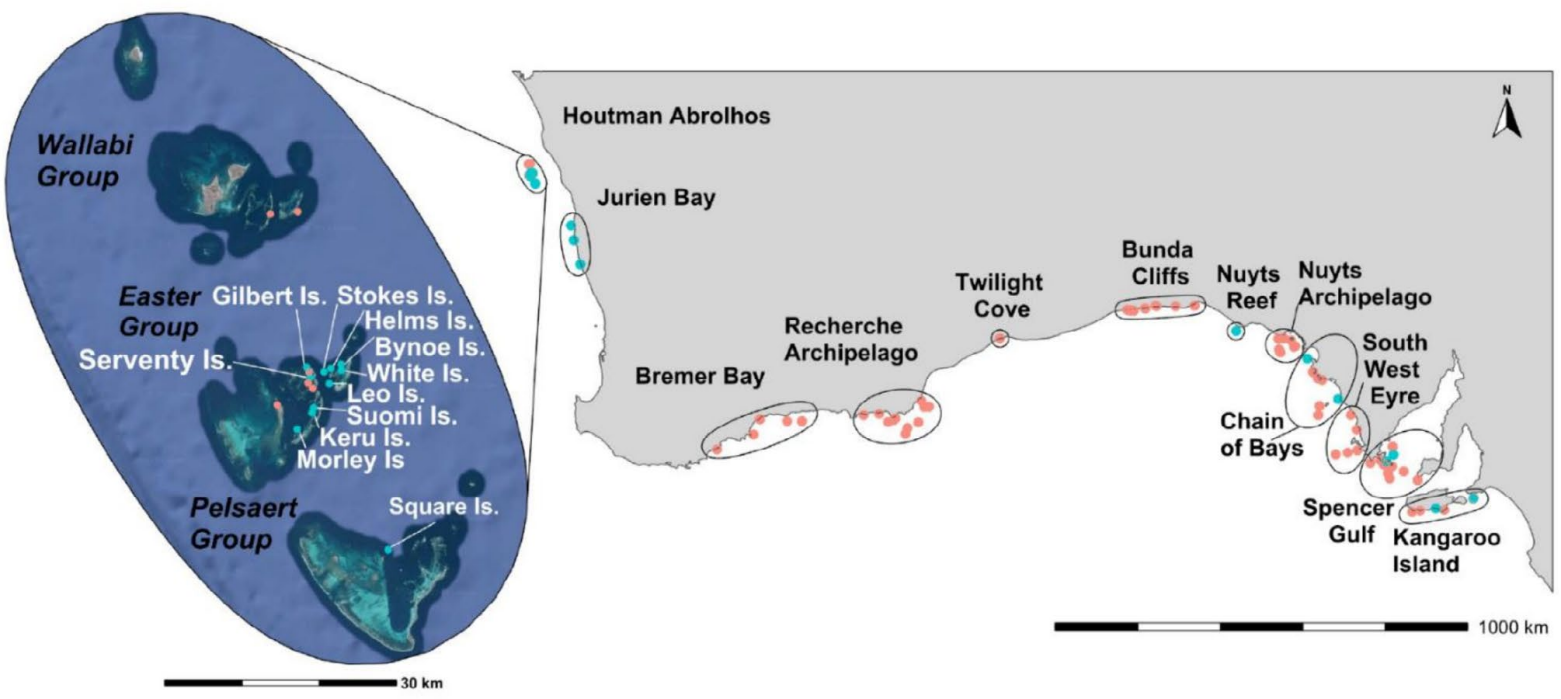
Map of south‐west Australia (right) and satellite imagery of the Houtman Abrolhos Islands (left). Sites inclusive of and West of Twilight Cove are in Western Australia. Sites east of Twilight Cove are in South Australia. Blue dots are sites sampled within this study. Red dots are unsampled, extant breeding sites within 12 geographical regions identified by Goldsworthy et al. (2021). GPS coordinates are published in table 1: Goldsworthy et al. (2021).

The Australian sea lion ( *Neophoca cinerea* ) (Figure 1) is Australia's only endemic and endangered seal species. Recent population trends indicate the ongoing decline of this species (Goldsworthy et al. 2021). Populations are dispersed across ~3000 km of coastline: South Australia (SA) is habitat to 82% of the species pup production at 48 extant breeding locations, whereas Western Australia (WA) has 18% of the pup production across 32 breeding sites (Goldsworthy et al. 2021) (see distribution map, Figure 2). This species' life history poses substantial conservation and management challenges, compounding the effect of climate change threats. Australian sea lions have multiple traits which may impede migration or redistribution; both sexes are philopatric (Ahonen et al. 2016); the species has a long and asynchronous breeding season between geographically close colonies (Gales et al. 1994); individuals maintain fidelity to benthic foraging sites throughout their lives (Lowther and Goldsworthy 2011); and females undertake relatively short duration foraging trips while caring for pups (~2 days) (Kirkwood and Goldsworthy 2013). Therefore, populations at the edge of the species' range, with limited neighbouring populations and in environments exposed to extreme climatic events, are likely to be the most vulnerable. Furthermore, population growth rates and size have been impacted by fisheries interactions (especially bycatch in demersal gillnet and rock lobster pots), entanglement in marine debris and disease (Goldsworthy et al. 2021, 2022, 2025). The Australian sea lion has the highest risk category rating for climate urgency due to multifaceted hazards (changes in habitat suitability, disease outbreaks and extreme weather events) and large knowledge gaps (Sojitra et al. 2024).

At the most northern breeding location of the species in WA, is an archipelago of approximately 150 low‐lying coral reef islands and islets extending over ~2500 km^2^, known as the Houtman Abrolhos Islands (hereafter referred to as the Abrolhos). The sea lions in this area were extensively hunted in the early 1800s (Campbell 2005). There has been some recovery since then, with the most recent counts (in 2023) estimating 120 individuals (S. Moore, pers. comm.). This temperate–tropical archipelago in the Indian Ocean was formed when sea level rose due to major global ice‐melt across the Pleistocene–Holocene transition, ~15,000 to 7000 years ago (Collins et al. 2004). The geographical isolation and fragmented formation of atolls and islands likely contributed to available habitat for sea lions in the western WA region of their range. The Abrolhos are exposed to the Leeuwin Current, a warm, low salinity, nutrient‐poor, continuous boundary current flowing southward along WA's coastline (Akhir et al. 2020). The Leeuwin Current has been exposed to warming *La Niña* events, including a pronounced heatwave event in 2011 (Pearce et al. 2011). That heatwave correlated with the death, contraction and southward redistribution of numerous fish and invertebrate communities (Pearce et al. 2011; Poloczanska et al. 2016). Consequently, global warming and its associated marine heatwaves are likely to negatively impact the resident Australian sea lions.

Examining the population genetics of the Abrolhos group of Australian sea lions is critical for understanding this population's vulnerability to historic and current pressures. Genetic structure of the Abrolhos group was assessed via mitochondrial DNA (mtDNA) over 30 years ago (Campbell, Gales, et al. 2008). This is difficult to compare with populations elsewhere, which have been assessed using nuclear markers such as microsatellites or single‐nucleotide polymorphisms (SNPs) (Lowther et al. 2012; Ahonen et al. 2016; Bilgmann et al. 2021). mtDNA analyses revealed shared haplotypes with breeding colonies ~200 km south, in the Jurien Bay region of WA, suggesting some female recruitment was occurring from sites outside of the Abrolhos (Campbell, Gales, et al. 2008). Advancements using SNPs provide a biparental marker of diversity, an indication of more recent genetic exchange (Kivisild 2015), and higher resolution for detecting population differentiation in comparison to mtDNA (Lah et al. 2016).

Given the geographic isolation of the Abrolhos Australian sea lions, the species' life history, historic and potential threats and increasing pressures of climate change, we hypothesise this group of animals is genetically isolated. To test this, we sampled pups from breeding colonies across SA and WA and completed SNP analysis. We aim to uncover genetic structure and diversity of the Abrolhos group of Australian sea lions located at the western and northern limit of the species' range, relative to other populations, important information which will benefit their conservation and management. Given the group's location and life history, we hypothesise the Abrolhos group will be (1) genetically distinct, reflecting low connectivity with other groups; and (2) have lower genetic diversity relative to groups of sea lions sampled at other locations.

## 2 Methods

### 2.1 Study Area and Sampling

Tissue samples were collected from 150 Australian sea lion pups (138 alive, 12 dead), across remote islands within WA and SA between February 2022–May 2023. Most pups were not fully moulted (< 6 months old) and therefore not able to disperse between colonies, optimising confidence that those sampled were born at the sites visited. Five pups were approximately 6–18 months old and unlikely to have been able to disperse. We sampled from 21 islands in total. In SA, we sampled from seven breeding colonies: Nuyts Reef north, West Waldegrave Island, Olive Island, Lewis Island, Dangerous Reef, Seal Bay on Kangaroo Island, and The Pages south. In WA, we sampled from three breeding colonies in the area of Jurien Bay: Beagle Island, North Fisherman Island, and Buller Island, and 11 islands from Abrolhos: Square Island (southern Pelsaert Group) and Gilbert, Serventy, Morley, Large Helms, Stokes, White, Suomi, Keru, South Bynoe, and Leo Islands from the Easter Group (Figure 2). These sample sites were chosen to compare genetic diversity and structure within and between geographical locations. All breeding locations within the Abrolhos were targeted given each location's small pup cohort. Logistical constraints restricted sampling from breeding colonies on the south coast of WA.

Sampling was permitted by ethic permits from Macquarie University Animal Ethics Committee (2021/024) and Government of SA's Primary Industries and Regions Ethics Committee (#16/20), and Wildlife Permits from the SA Department for Environment and Water (A27144 and A24684) and WA Department of Biodiversity, Conservation and Attractions (TFA 2022‐0020).

### 2.2 Tissue Collection

Live pups were caught by hand or net when the mother was absent. We collected 1–5 mm of skin tissue from the hind flipper using a sterile scalpel blade or biopsy punch. Fur was clipped from each sampled pup's back so it could be recognised to avoid resampling. Tissue was immediately stored in a 2 mL microcentrifuge tube with 95%–100% ethanol. During fieldwork and travel, samples were stored in a cool box with ice packs or in a freezer at −15°C where available until they could be stored at −30°C until analysis.

### 2.3 DNA Extraction and Sequencing

A total of 150 tissue samples, plus duplicates of 23 individuals (resulting in a total of 173 samples), were sent to Diversity Arrays Technology Pty. Ltd. (DArT, Canberra, Australia) for DNA extraction, DArTseq sequencing and SNP genotyping. DArTseq is a next‐generation sequencing approach using complexity reduction via restriction enzymes PstI (5′‐CTGCA|G‐3′) and SphI (5′‐GCATG|C‐3′), which target non‐repetitive regions of the genome. Sequencing was performed on an Illumina NovaSeq 6000 and NovaSeq X Plus. All data were generated as single‐end reads with a read length of 138 bp. SNP genotyping was conducted using proprietary DArT Pty Ltd. analytical pipelines. DArT's technology is reference genome independent. More details of the DArT process are provided in Georges et al. (2018). After initial filtering by DArT, 18,297 SNP loci were retained from 163 samples representing 142 pups. Samples from five WA individuals and three SA individuals were discarded due to low concentration DNA most likely linked to tissue quality.

### 2.4 SNP Calling and Data Filtering

Prior to additional filtering specific to our aims and study design, we used the biological replicates (19, as four failed) to detect mismatched loci (mean error rate = 4.51%; Table S1) in R v4.3.3 (R Core Team 2021); 1063 SNPs mismatched in two or more replicates and were removed from the whole dataset. If one replicate sample had a call rate lower than 80%, it was removed from subsequent analysis. If both replicates had a call rate higher than 80%, one replicate was randomly chosen per individual.

Further filtering was conducted using the R package ‘dartR’ (Gruber et al. 2018) in the following order. Eight samples, which had very low genotyping call rates (< 35%) were removed. A further 11 samples considered to be low quality (call rate < 68%) were also removed. We retained as many Abrolhos individuals as possible so as to not compromise the primary objective of our study design, which was centred on this range‐limit archipelago. Thresholds for the following filter steps were chosen to retain useful loci and minimise the error rate by removing lower quality data without over filtering. We determined thresholds by examining ‘genlight reports’ and smear plots of the dataset (Gruber et al. 2018). We removed SNPs with a low repeatability count (< 90% reproducibility) and high missing data (< 80% call rate by loci). To reduce the likelihood of false homozygous calls, we retained SNPs with high number sequence reads across all individuals (> 3 and < 13 rdepth). We removed secondary SNPs within sequencing fragments, and loci with minor allele frequency (MAF) < 0.012% (i.e., alleles seen at least three times in the dataset, in at least two individuals, were retained [3/2 N]) (Linck and Battey 2019). We tested for sex‐linked loci (Robledo‐Ruiz et al. 2023) and found no strong evidence that any of the remaining loci were sex linked. SNPs that significantly (at *α* = 0.05) deviated from Hardy Weinberg equilibrium in at least half of the populations (where *n* > 5) were removed from the entire dataset. The final dataset contained 2288 SNPs for analysis from 125 individuals from 19 sample sites (see Table S2 for sample sizes for each sample site post filtering).

### 2.5 Population Structure

We investigated Australian sea lion population structure using three clustering methods. To visualise genetic similarity between individuals, we used an unsupervised, distance‐based (Euclidean), principal coordinate analysis (PCoA) following the protocols of Georges et al. (2023). Firstly, we analysed all individuals together and then we subset the data to sample sites from within WA (38 pups, sample sites = 12) and SA (87 pups, sample sites = 7) to determine if there were nested groups or sub‐structuring.

To visualise genetic similarity among sample sites we constructed an unrooted phenetic tree using a neighbour‐joining approach (or unweighted pair group method analysis UPGMA). This method converts the data to a matrix of frequencies (locus by populations) and computes Euclidean distances between groups of closely related individuals to produce a phylogram.

To determine whether individuals from sample sites represent distinct genetic groups, we used an unsupervised, Bayesian model‐based clustering algorithm, STRUCTURE v2.3 (Falush et al. 2003; Mijangos et al. 2022). STRUCTURE was run 10 times for each possible number of populations (*K*), ranging from 2:19 (total number of sampled colonies) using a burn‐in period of 50,000 with 100,000 repetitions of Markov Chain Monte Carlo (MCMC) iterations, without admixture (following Pritchard et al. 2010; Porras‐Hurtado et al. 2013). We ran the function without admixture, as this option is more powerful for detecting subtle structure (Pritchard et al. 2010). Instead of using the default model, alternative ancestry model parameters were used because sample sizes among sites varied (*n* = 1 to 17) and Australian sea lions are known to have closely related individuals within colonies (Campbell, Gales, et al. 2008). The parameter POPALPHAS = 1 was used to account for unbalanced sampling among sample sites (Wang 2022) and LOCPRIOR = 1 to account for small sample sizes and close relationships between some pairs of sample sites (Porras‐Hurtado et al. 2013). The optimal number of clusters was inferred using the Evanno method which is based on the rate of change in log probability (Δ*K*) among consecutive *K*‐values (Evanno et al. 2005). Then, the optimal number of clusters inferred from Δ*K* was used to aggregate and visualise individuals into that number of genetic clusters via the CLUMPAK method (Kopelman et al. 2015). To establish whether there was evidence of population subdivision within WA, subsequent hierarchical analysis was completed, as recommended by Pritchard et al. (2010) and Janes et al. (2017), using the within WA data subset. We used the same settings and methods as the full dataset analysis (adjusting range of (*K*) to 1–12 in STRUCTURE).

### 2.6 Measures of Genetic Differentiation and Diversity Between Identified Genetic Clusters

To evaluate gene flow between populations we calculated the fixation index (*F*_ST_) and performed 200 bootstraps for 95% confidence intervals. To quantify genetic diversity within identified populations, we measured expected heterozygosity (*H*_E_), observed heterozygosity (*H*_O_), and the population‐level inbreeding coefficient (*F*_IS_) and standard error of each.

## 3 Results

### 3.1 Population Structure

**FIGURE 3:**
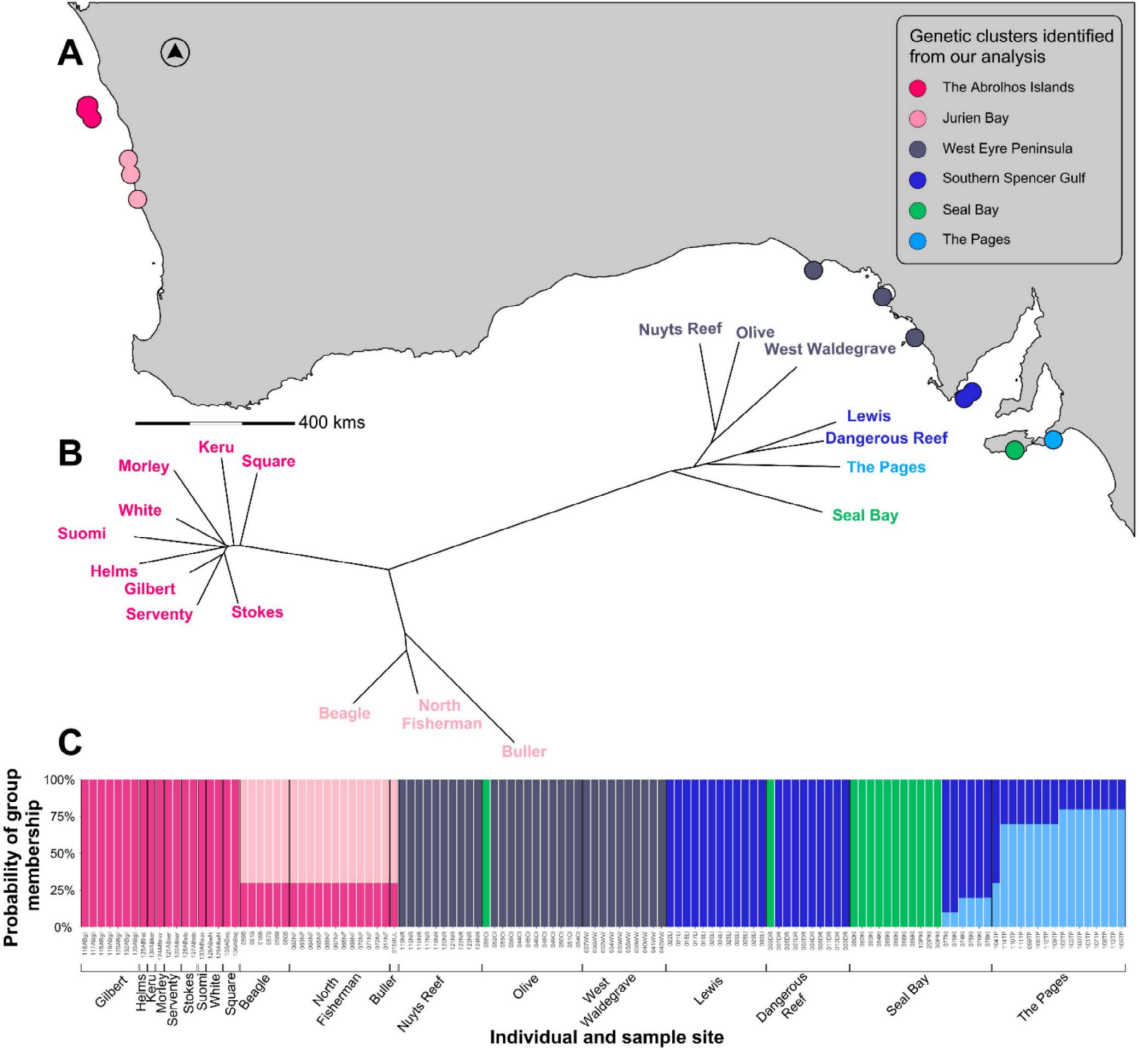
The map (A), dendrogram (B) and STRUCTURE analysis plot (C) show the upper level of six inferred genetic clusters from single nucleotide polymorphisms (SNPs) from 125 Australian sea lion pups sampled from 19 sample sites across different locations within Western Australia (WA) and South Australia (SA). Six colours are used to represent the six genetically distinct populations identified, named (from West to East) (1) the Abrolhos (magenta) and (2) Jurien Bay (light pink) in WA and (3) West Eyre Peninsula (dark grey), (4) Southern Spencer Gulf (royal blue), (5) Seal Bay (green) and (6) The Pages (light blue) in SA. (A) Map of sample site locations seven in SA, 12 in WA (three in Jurien Bay, nine in the Abrolhos). (B) Neighbour joining phenetic tree showing genetic distance between individuals from each sample site. (C) STRUCTURE analysis plot showing the probability of which cluster(s) individuals can be assigned to.

Australian sea lions showed strong genetic structure among geographical regions. The greatest differentiation was between WA and SA populations with sub‐structuring found within each region (Figure 3B and Figure S1a–c). Within WA, PCoA2 (Figure S1b) showed dissimilarity between individuals from the Abrolhos sample sites and individuals from Buller, Beagle and North Fisherman Islands. This result was also observed in the dendrogram, which had two distinct lines of ancestry (Figure 3B). STRUCTURE analysis assigned individuals into six genetic clusters across WA and SA (Δ*K* = 6) (Figure 3C, Figure S2, Table S3) and into two genetic clusters within WA in both the full dataset (Figure 3C) and WA only analysis (Δ*K* = 2) (Figures S3 and S4, Table S4).

Within WA, all individuals sampled from the Abrolhos were assigned to one distinct genetic cluster (Figure 3B,C and Figure S1a,b). All individuals from Jurien Bay sites (North Fisherman, Buller and Beagle Islands) were assigned to a second cluster with 30% probability of membership to the cluster to which the Abrolhos pups were assigned. SA individuals were assigned exclusively or partly to one of four clusters. Out of 12 Olive Island individuals, 11 clustered with all West Waldegrave Island and Nuyts Reef individuals. Nine out of 10 Dangerous Reef individuals clustered with all Lewis Island individuals. Most individuals sampled from The Pages (*n* = 15/16) were assigned primarily to a unique cluster (with 70%–80% assignment) and partially assigned to the Dangerous Reef/Lewis Island cluster (20%–30% assignment). One individual from The Pages assigned mostly to the Dangerous Reef/Lewis Island cluster (70% probability, 30% to The Pages cluster). Of Seal Bay individuals, 65% (*n* = 11/17) were assigned to a unique cluster, while the other 35% clustered mostly (80%–90% probability) with the Dangerous Reef/Lewis Island cluster individuals and partially (10%–20% probability) with The Pages' unique cluster. One Olive Island and one Dangerous Reef pup both assigned (100% probability) to the unique Seal Bay cluster.

Considering the three analyses methods, we suggest six genetic clusters, in geographical order from West to East: (1) all nine islands from the Abrolhos, (2) Beagle, North Fisherman and Buller Islands, (3) Nuyts Reef, Olive and West Waldegrave Islands, (4) Lewis Island and Dangerous Reef, (5) Seal Bay and (6) The Pages. Hereafter we will refer to each as populations: (1) the Abrolhos, (2) Jurien Bay, (3) West Eyre Peninsula, (4) Southern Spencer Gulf, (5) Seal Bay and (6) The Pages.

The highest degree of genetic differentiation among the six genetic clusters was between the Abrolhos and The Pages, which are at the north‐western and south‐eastern fringes of the species' range, respectively (Table 1). The two populations within WA, the Abrolhos and Jurien Bay, were highly differentiated from each other and from all other clusters in SA. Within SA, Seal Bay was genetically differentiated from Western Eyre Peninsula and The Pages but not from Southern Spencer Gulf. The Pages, Spencer Gulf and Western Eyre Peninsula had a low level of differentiation between populations.

**TABLE 1:** Pairwise genetic differences (*F*_ST_) (95% CI) calculated from single nucleotide polymorphisms (SNPs) for the six Australian Sea Lion populations from South Australia (SA) and Western Australia (WA) identified from our structure analysis.

	The Abrolhos (WA)	Jurien Bay (WA)	Western Eyre Peninsula (SA)	Southern Spencer Gulf (SA)	Seal Bay (SA)
Jurien Bay (WA)	0.564 (0.524–0.599)				
Western Eyre Peninsula (SA)	0.589 (0.575–0.607)	0.469 (0.454–0.485)			
Southern Spencer Gulf (SA)	0.619 (0.604–0.634)	0.489 (0.474–0.507)	0.076 (0.069–0.082)		
Seal Bay (SA)	0.621 (0.605–0.638)	0.564 (0.548–0.579)	0.178 (0.166–0.189)	0.143 (0.132–0.153)	
The Pages (SA)	0.679 (0.665–0.697)	0.542 (0.522–0.559)	0.120 (0.112–0.127)	0.082 (0.075–0.089)	0.164 (0.151–0.175)

### 3.2 Genetic Diversity

Australian sea lions sampled in SA had a markedly higher level of gene diversity (probability that alleles will be heterozygous) (*H*_E_ = 0.20–0.22) compared to those in WA (*H*_E_ = 0.02–0.07) (Table 2). Individuals from the Abrolhos had the lowest proportion of heterozygous individuals (*H*_O_ = 0.02), whereas individuals from the Southern Spencer Gulf had the highest (*H*_O_ = 0.20), 10 times more than the Abrolhos. Seal Bay had the highest level of population‐level inbreeding (*F*_IS_ = 0.27), followed closely by the Abrolhos (*F*_IS_ = 0.23), suggesting mating within these populations is not random, whereas Jurien Bay was close to zero (the lowest level across all populations; *F*_IS_ = 0.06) (Table 2).

**TABLE 2:** Genetic diversity statistics calculated from single nucleotide polymorphisms (SNPs) for the six Australian Sea Lion populations from South Australia (SA) and Western Australia (WA) identified from our structure analysis.^a^

	*n*	*H*_E_ (SE)	*H*_O_ (SE)	*F*_IS_ (SE)
The Abrolhos (WA)	19	0.023 (0.002)	0.016 (0.001)	0.230 (0.008)
Jurien Bay (WA)	19	0.072 (0.003)	0.068 (0.003)	0.058 (0.005)
Western Eyre Peninsula (SA)	32	0.217 (0.004)	0.184 (0.004)	0.170 (0.006)
Southern Spencer Gulf (SA)	22	0.218 (0.004)	0.200 (0.004)	0.119 (0.006)
Seal Bay (SA)	17	0.219 (0.004)	0.156 (0.004)	0.272 (0.008)
The Pages (SA)	16	0.201 (0.004)	0.172 (0.004)	0.155 (0.007)

^a^ *n* = sample size, HE = expected heterozygosity, HO = observed heterozygosity, FIS = population‐level inbreeding coefficient, SE = standard error.

## 4 Discussion

In this study we assessed the genetic connectivity of Australian sea lions from the most north‐westerly breeding location of the species, the Abrolhos Islands, which has been exposed to historic and current threats. The Abrolhos population is genetically isolated from other studied populations across the sea lion's range, including within WA, and found to have very low levels of genetic diversity. Given the Australian sea lion's specialised land‐sea requirements, natal site fidelity (Campbell, Gales, et al. 2008; Lowther et al. 2012), and the increasing influence of climate change, the long‐term survival of the Abrolhos population is uncertain and requires a different management strategy to other populations across the species range.

Our findings clearly illustrate that the west coast WA populations of Australian sea lions are distinct from SA populations. Importantly, the Abrolhos is distinct from its nearest population in Jurien Bay (Figure 3 and Table 1). This indicates that there is restricted migration of animals between these two populations despite being only 200 km apart. Based on mtDNA collected in 1989 and 1999 (Campbell 2003), a single matriline (fixed haplotype) linked pups from both the Abrolhos and Jurien Bay (Campbell, Gales, et al. 2008). This suggested that both populations are descendants from the same females. Campbell, Gales, et al. (2008) predicted that biparental inherited DNA, for example that obtained using microsatellite and SNP markers, would show a minimal level of population subdivision. This reflects patterns found across many pinniped species, for example the Galapagos fur seal ( *Arctocephalus galapagoensis* ), where philopatric females and dispersing males cause a pronounced structure with mtDNA but a weak structure with microsatellite markers (Lopes et al. 2015). In contrast, using SNPs we found a substantial differentiation (pairwise *F*_ST_ = 0.56) between the Abrolhos and Jurien Bay populations, indicating that both male and female individuals contribute to the high level of structure (Flanagan and Jones 2019). Data from mtDNA is useful for historical lineage tracing, but can be confounded by homoplasy, whereas SNPs have higher resolution for detecting recent gene flow and subtle population structure (Kraft et al. 2020). Both previous and current findings together suggest that the Abrolhos and Jurien Bay populations share a historical maternal lineage with minimal genetic exchange recently. Investigating estimated divergence times could further clarify admixture observed across all Jurien Bay individuals (30% of Abrolhos but not *vice versa*). Although our study covers samples from across the species' distribution, gaps remain located in south WA coast and western SA (Figure 2).

Lack of gene flow into the Abrolhos sea lion population may result from numerous interacting factors both historic and contemporary. The population faced commercial sealing between the 16th and 20th centuries, significantly depleting sea lion numbers (Campbell 2005). The Abrolhos population is currently threatened by fisheries interactions, human disturbance and disease (Campbell, Holley, et al. 2008; Goldsworthy et al. 2025; Department of Sustainability, Environment, Water, Population and Communities 2013; Department of Biodiversity, Conservation and Attractions 2023). Climate change is now also causing serious concern affecting prey availability, an increase in temperature and inundation (sea level rise and storms) (Schumann et al. 2013), with marine heatwaves in the region in 2011 and 2025 (Pearce et al. 2011; Wernberg et al. 2016; Department of Primary Industries and Regional Development 2025). Australian sea lions, unique among otariids, lack the ability to adjust their metabolism in response to warming waters (Ladds et al. 2017), which may further impair their physical ability to forage and reproduce in the face of climate change.

An underpinning paradigm of conservation biology is that reduced genetic diversity results in the lack of capacity to adapt to environmental change (Madsen et al. 1999; Spielman et al. 2004; Lai et al. 2019). We found relatively higher diversity in the SA populations, which is indicative of their higher abundance and connectivity (Davis and Shaw 2001). In contrast, genetic diversity was an order of magnitude lower in the Abrolhos population (*H*_O_ = 0.016), with diversity also relatively low within the closest WA population, Jurien Bay (*H*_O_ = 0.068) (Table 2). Peripheral populations are found to have low genetic diversity due to small population sizes and limited immigration from other populations (Eckert et al. 2008). This can be attributed to fringe populations starting with a smaller number of individuals to establish the population and experiencing unidirectional connectivity (Signorile et al. 2014; Ramos et al. 2018). In comparison to the Jurien Bay population, the Abrolhos population is smaller and spread out across many island groups. Around Jurien Bay, the waters are managed as a marine park, whereas at the Abrolhos, the waters and marine life are not protected. The isolated location and low population density, in combination with past hunting, minimal management and other undocumented pressures, likely collectively contribute to the low genetic diversity we find for the Abrolhos population. A primary concern for populations with low levels of genetic diversity is their capacity to respond to any stochastic, catastrophic changes and reduced potential to recover from such events elevating extinction risk (Spielman et al. 2004).

Geographical isolation can drive local adaptation (Bernaś et al. 2021). While yet to be documented in the case of the Abrolhos Australian sea lion population, local prey‐driven adaptations were found in isolated colonies of Antarctic fur seal ( *Arctocephalus gazella* ) (Cleary et al. 2019) and female Guadalupe fur seals ( *Arctocephalus townsendi* ) expanded their prey preference post a warming event (Amador‐Capitanachi et al. 2020). Evidence from SA shows that even over a small spatial scale, Australian sea lions exhibit niche foraging patterns (Lowther and Goldsworthy 2011), suggesting there is a link between community structure and foraging. It is possible the Abrolhos population may have already adapted to survive their niche environment. Adaptation to higher temperatures is a possible response to warmer and variable marine environments as sea lions are unlikely to shift geographical location (Harvey et al. 2022), albeit Australian sea lions may be more vulnerable than other species (see above). For example, Galapagos sea lions ( *Zalophus wollebaeki* ) and Galapagos fur seals have adapted to survive a tropical, variable climate (Riofrío‐Lazo and Páez‐Rosas 2021). If WA populations have a functional variation beneficial for climate adaptation, this could reduce their vulnerability to warming environments (Booth et al. 2024). Future research investigating whether adaptations have occurred between SA and WA populations could help determine whether the Abrolhos population has ecologically important genetic variations and would provide further evidence into the populations' resilience to future environmental change (Meek et al. 2023).

Given that we sampled ~90% of the Abrolhos recruits (S. Moore, pers. comm.) we are confident with the findings of our study which highlights the vulnerability of this Australian sea lion population. Targeted conservation actions are needed urgently to protect the population. Genetic rescue via translocation is an important management option to boost genetic variation (Whiteley et al. 2015); however, the species' philopatric behaviour and the risk of outbreeding depression must be considered (Ralls et al. 2018). Limiting the impact of cumulative local threats is vital for survival (Nielsen et al. 2023) and should be the priority for animals in the Abrolhos region. While taking into account issues such as disease and fishery interactions, the effects of marine heatwaves need to be taken into consideration. This might include actions such as habitat restoration or heat shelters for pups, particularly if predicted events will coincide with the breeding season. Our study reflects the increasing vulnerability of animals at the edge of their distributions, particularly those losing resources due to a rapidly warming environment and further magnified where population genetics has eroded.

## Author Contributions

**Vanessa Morris:** conceptualization (equal), data curation (lead), formal analysis (lead), funding acquisition (lead), investigation (lead), project administration (lead), software (lead), visualization (lead), writing – original draft (lead), writing – review and editing (lead). **Anthony Chariton:** conceptualization (equal), funding acquisition (equal), resources (equal), supervision (equal), writing – original draft (supporting), writing – review and editing (equal). **Robert Harcourt:** conceptualization (equal), funding acquisition (equal), supervision (equal), writing – original draft (supporting), writing – review and editing (equal). **Catherine E. Grueber:** formal analysis (supporting), software (supporting), supervision (supporting), writing – original draft (supporting), writing – review and editing (equal). **Isabelle Charrier:** funding acquisition (equal), supervision (supporting), writing – review and editing (equal). **Holly Raudino:** investigation (supporting), writing – review and editing (equal). **Kelly Waples:** investigation (supporting), writing – review and editing (equal). **Roger Kirkwood:** investigation (supporting), writing – review and editing (equal). **Simon D. Goldsworthy:** investigation (supporting), writing – review and editing (equal). **Benjamin J. Pitcher:** conceptualization (equal), funding acquisition (equal), investigation (equal), supervision (equal), writing – original draft (supporting), writing – review and editing (equal).

## Funding

This work was supported by the Australian Sea Lion Recovery Foundation and Centre National de la Recherche Scientifique.

## Conflicts of Interest

The authors declare no conflicts of interest.

## Supporting information


**Data S1:** ece373038‐sup‐0001‐Supinfo.docx.

## Data Availability

The dataset is publicly accessible at this link: https://doi.org/10.5061/dryad.3ffbg79xw.
