# Supplementary material for: Population Structure and Genetic Diversity Analyses Reveal Isolation That May Imperil the Northernmost Colony of the Endangered Australian Sea Lion
Source: Ecol Evol. 2026 Feb 4;16(2):e73038. doi: 10.1002/ece3.73038 (PMC12873503; doi:10.1002/ece3.73038)
Supplement: Supplementary file 1 — Data S1: ece373038‐sup‐0001‐Supinfo.docx. [file ECE3-16-e73038-s001.docx]

**Supporting information to:**

**Population structure and genetic diversity analyses reveal isolation that may imperil the northernmost colony of the endangered Australian sea lion.**

**SI Table 1** Biological replicates used to detect mismatched loci.

| PairID | First | Second | called.loci | match | error |
| --- | --- | --- | --- | --- | --- |
| 1 | 116ABgil230509 | 116ABgil230509R | 7139 | 94.77518 | 5.224821 |
| 2 | 117ABgil230509 | 117ABgil230509R | 12569 | 96.47546 | 3.524545 |
| 3 | 119ABgil230509 | 119ABgil230509R | 12228 | 95.87831 | 4.121688 |
| 4 | 120ABgil230509 | 120ABgil230509R | 14932 | 96.79882 | 3.201179 |
| 5 | 121ABser230509 | 121ABser230509R | 13626 | 96.80023 | 3.199765 |
| 6 | 122ABser230509 | 122ABser230509R | 11383 | 95.8359 | 4.164104 |
| 7 | 124ABmor230509 | 124ABmor230509R | 13081 | 96.62105 | 3.378947 |
| 8 | 125ABhel230510 | 125ABhel230510R | 12283 | 96.32012 | 3.679883 |
| 9 | 126ABsto230510 | 126ABsto230510R | 13375 | 95.94766 | 4.052336 |
| 10 | 127ABsto230510 | 127ABsto230510R | 12678 | 95.3936 | 4.606405 |
| 11 | 128ABwhi230510 | 128ABwhi230510R | 14190 | 96.31431 | 3.685694 |
| 12 | 129ABwhi230510 | 129ABwhi230510R | 14378 | 96.68939 | 3.310613 |
| 13 | 130ABgil230511 | 130ABgil230511R | 13957 | 96.33159 | 3.66841 |
| 14 | 132ABgil230511 | 132ABgil230511R | 5273 | 94.27271 | 5.72729 |
| 15 | 133ABsuo230511 | 133ABsuo230511R | 3270 | 90.55046 | 9.449541 |
| 16 | 134ABker230511 | 134ABker230511R | 12999 | 96.2074 | 3.792599 |
| 17 | 135ABsq230512 | 135ABsq230512R | 3153 | 89.94608 | 10.05392 |
| 18 | 136ABsq230512 | 136ABsq230512R | 14200 | 96.71127 | 3.288732 |
| 19 | 060BI220601 | 060BI220601R | 16503 | 96.41277 | 3.587227 |
|  |  | **Mean** | 11643 |  | 4.511458 |

**SI Table 2** Sample size for sample site post DNA extraction, SNP calling and data filtering.

| Sample site | Data analysis  sample size |
| --- | --- |
| Gilbert Island | 7 |
| Helms Island | 1 |
| Keru Island | 1 |
| Morley Island | 1 |
| Serventy Island | 2 |
| Stokes Island | 2 |
| Suomi Island | 1 |
| White Island | 2 |
| Square Island | 2 |
| Beagle | 6 |
| North Fisherman | 12 |
| Buller | 1 |
| Nuyts Reef | 10 |
| Olive Island | 12 |
| West Waldegrave | 10 |
| Lewis Island | 12 |
| Dangerous Reef | 10 |
| Kangaroo Island | 17 |
| The Pages | 16 |


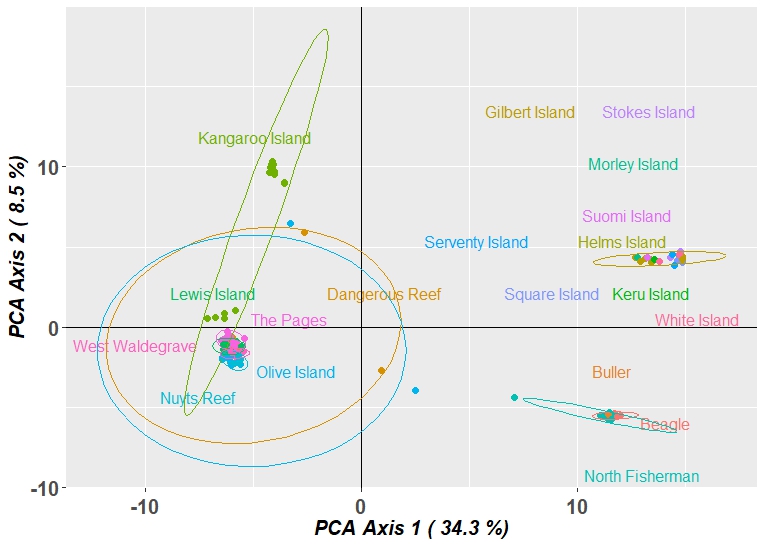


**SI Figure 1a** PCoA #1 showing axis 1 and 2 of all Australian sea lion samples from all sampled sites (population ellipses are 95% percentile).


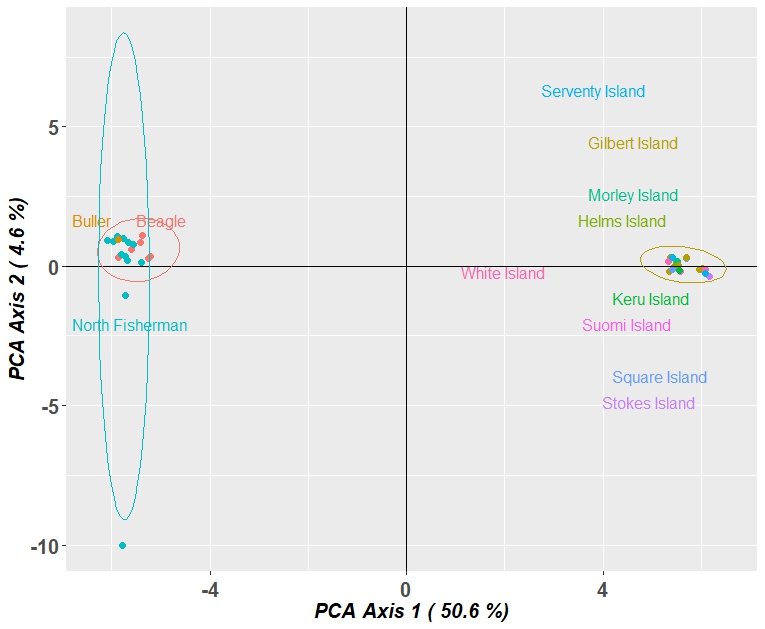


**SI Figure 1b** PCoA #2 of Australian sea lion samples from Western Australia sample sites only (population ellipses are 95% percentile).


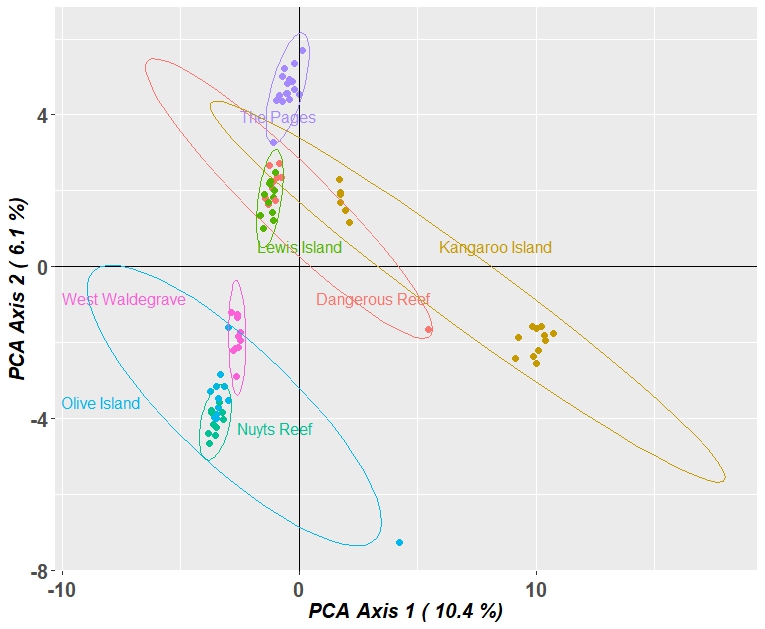


**SI Figure 1c** PCoA #3 of Australian sea lion samples from South Australia sample sites only (population ellipses are 95% percentile).


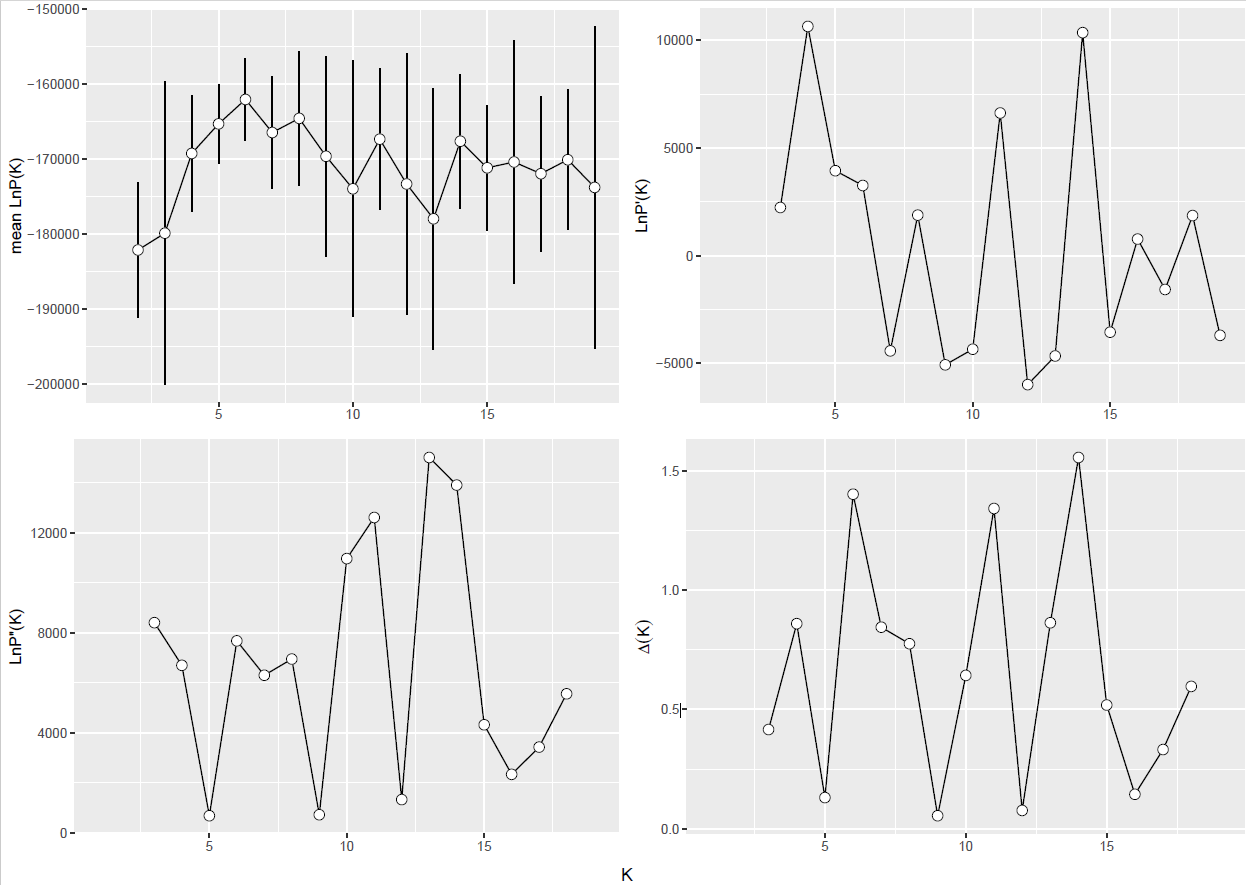


**SI Figure 2** Evanno plots showing posterior probability used to detect the number of K groups which best fit the data for Australian sea lion samples from all sampled sites. Top left panel: Mean likelihood of posterior probability for each K. Top right panel: mean difference between successive likelihood values of K (LnP’(K)). Bottom left panel: absolute value of the difference between successive values of LnP’(K) referred to as LnP”(K). Bottom right panel: estimate delta K, the mean of the absolute values of LnP”(K).

**SI Table 3** Evanno/Posterior probability values corresponding to SM Figure 2. The row highlighted green represents the initial highest delta K value and therefore corresponding number of k groups.

| k | reps | mean.ln.k | sd.ln.k | ln.pk  (LnP’(K)) | ln.ppk (LnP”(K)) | delta.k |
| --- | --- | --- | --- | --- | --- | --- |
| 2 | 10 | -182132 | 8946.893 | NA | NA | NA |
| 3 | 10 | -179892 | 20216.13 | 2240.76 | 8404.84 | 0.415749 |
| 4 | 10 | -169246 | 7792.163 | 10645.6 | 6697.81 | 0.859557 |
| 5 | 10 | -165298 | 5261.109 | 3947.79 | 686.13 | 0.130415 |
| 6 | 10 | -162037 | 5475.007 | 3261.66 | 7677.01 | 1.402192 |
| 7 | 10 | -166452 | 7460.235 | -4415.35 | 6301.33 | 0.844656 |
| 8 | 10 | -164566 | 8963.018 | 1885.98 | 6949.32 | 0.775333 |
| 9 | 10 | -169629 | 13331.82 | -5063.34 | 725.69 | 0.054433 |
| 10 | 10 | -173967 | 17062.32 | -4337.65 | 10965.01 | 0.642645 |
| 11 | 10 | -167340 | 9393.679 | 6627.36 | 12609.26 | 1.342313 |
| 12 | 10 | -173321 | 17391.48 | -5981.9 | 1331.03 | 0.076533 |
| 13 | 10 | -177972 | 17385.18 | -4650.87 | 15007.91 | 0.863259 |
| 14 | 10 | -167615 | 8935.174 | 10357.04 | 13904.19 | 1.556118 |
| 15 | 10 | -171162 | 8338.427 | -3547.15 | 4322.3 | 0.518359 |
| 16 | 10 | -170387 | 16235.94 | 775.15 | 2339.41 | 0.144088 |
| 17 | 10 | -171952 | 10336.37 | -1564.26 | 3429.78 | 0.331817 |
| 18 | 10 | -170086 | 9320.816 | 1865.52 | 5560.06 | 0.596521 |
| 19 | 10 | -173781 | 21492.97 | -3694.54 | NA | NA |


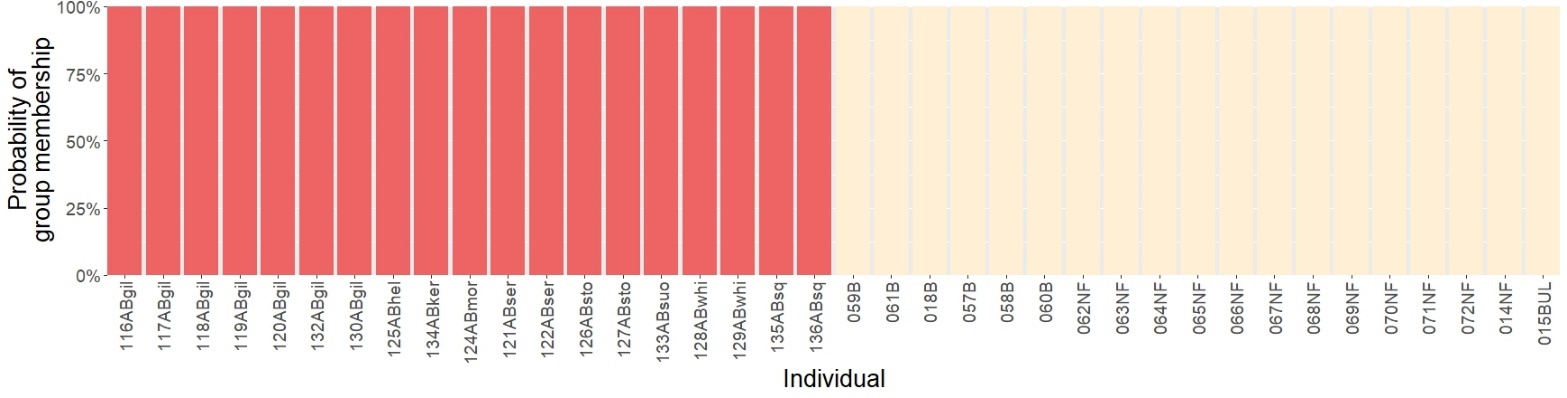


**SI Figure 3** STRUCTURE analysis plot showing the probability of which cluster(s) individuals sampled from Western Australia only can be assigned to. All individuals sampled from the Abrolhos region cluster into one group (orange/red colour), whereas all individuals sampled from the Jurien Bay region area cluster into a second group (cream colour).


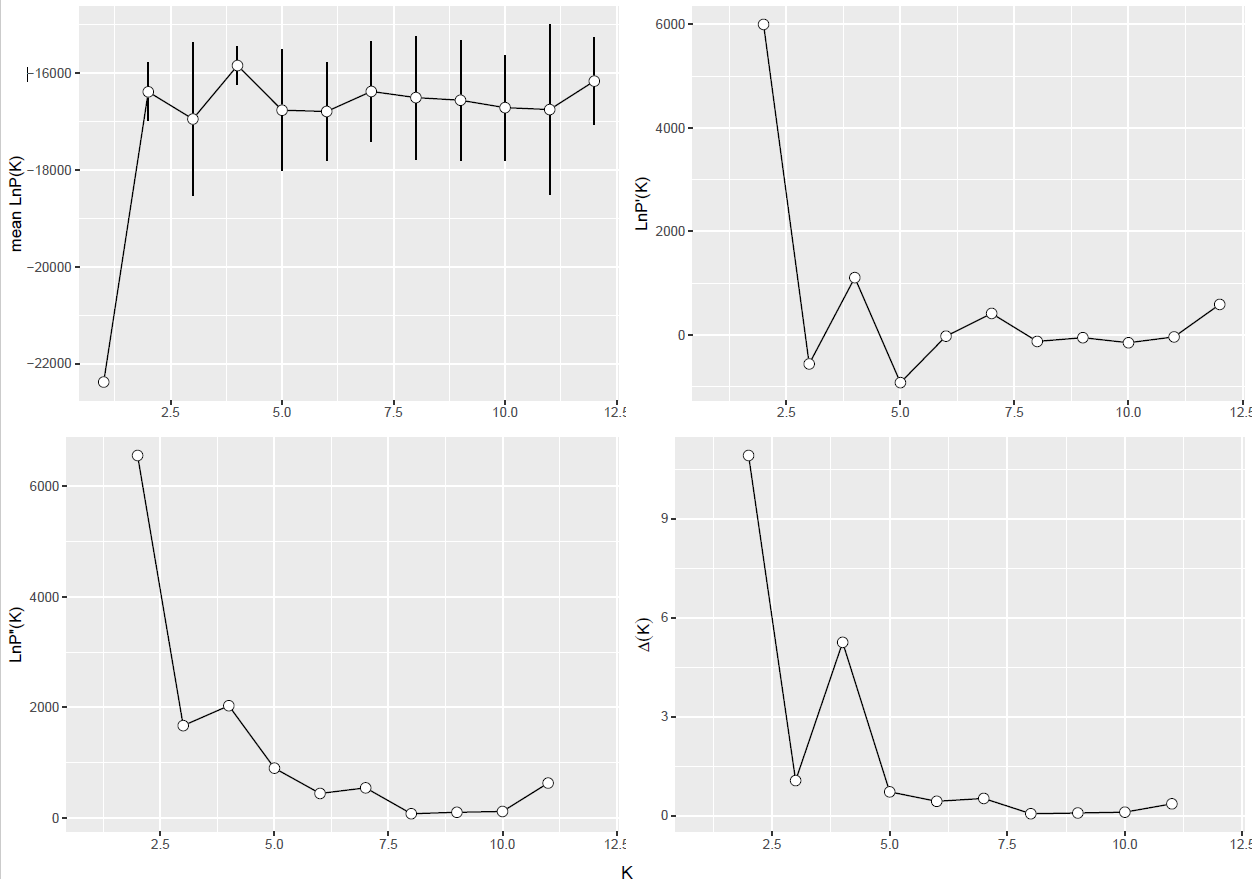


**SI Figure 4** Evanno plots showing posterior probability used to detect the number of K groups which best fit the data for Australian sea lion samples from Western Australia sampled sites. Top left panel: Mean likelihood of posterior probability for each K. Top right panel: mean difference between successive likelihood values of K (LnP’(K)). Bottom left panel: absolute value of the difference between successive values of LnP’(K) referred to as LnP”(K). Bottom right panel: estimate delta K, the mean of the absolute values of LnP”(K).

**SI Table 4** Evanno values corresponding to SM Figure 4. The row highlighted green represents the initial highest delta K value and therefore corresponding number of k groups.

| k | reps | mean.ln.k | sd.ln.k | ln.pk  (LnP’(K)) | ln.ppk (LnP”(K)) | delta.k |
| --- | --- | --- | --- | --- | --- | --- |
| 1 | 10 | -22380.9 | 11.9705 | NA | NA | NA |
| 2 | 10 | -16381.8 | 601.1538 | 5999.11 | 6561.68 | 10.91514 |
| 3 | 10 | -16944.4 | 1571.619 | -562.57 | 1668.1 | 1.06139 |
| 4 | 10 | -15838.9 | 386.4747 | 1105.53 | 2029.16 | 5.250435 |
| 5 | 10 | -16762.5 | 1247.787 | -923.63 | 898.04 | 0.719706 |
| 6 | 10 | -16788.1 | 1015.883 | -25.59 | 439.53 | 0.432658 |
| 7 | 10 | -16374.1 | 1039.719 | 413.94 | 541.16 | 0.520487 |
| 8 | 10 | -16501.4 | 1273.105 | -127.22 | 72.62 | 0.057042 |
| 9 | 10 | -16556 | 1243.666 | -54.6 | 98.18 | 0.078944 |
| 10 | 10 | -16708.7 | 1086.457 | -152.78 | 112.88 | 0.103897 |
| 11 | 10 | -16748.6 | 1760.202 | -39.9 | 627.71 | 0.356612 |
| 12 | 10 | -16160.8 | 902.9941 | 587.81 | NA | NA |
